# Supplementary material for: Socioeconomic level and associations between heat exposure and all-cause and cause-specific hospitalization in 1,814 Brazilian cities: A nationwide case-crossover study
Source: PLoS Med. 2020 Oct 8;17(10):e1003369. doi: 10.1371/journal.pmed.1003369 (PMC7544074; doi:10.1371/journal.pmed.1003369)
Supplement: S1 Text — (DOCX) [file pmed.1003369.s002.docx]

**S1 Text. Prospective analysis plan and modifications following comments from editors and reviewers**

**Date of commencing this research:** April 10th, 2019.

**Research type:** A time-stratifed case-crossover study

**Research objectives**

1. To quantify the associations between high ambient temperture (heat exposure) and all-cause and cause-specific hospitalizations.

2. To explore whether there is an inter-city socioeconomic disparity in the heat-hospitalization associations.

3. To explore the inter-city socioeconmic dispairty in heat-hospitalization vary by sex, age, and different cause-spefic hospitalizations.

**Hospitalization data**

Daily hospitalization cases recorded by Brazil’s National Unified Health System has been extracted for 1,814 cities in the hot seasons (city-specific four adjacent hottest months) during 2000−2015, as detailed by our previous study [1]. In this study, we will focus on all-cause hospitalizations and hospitalizations due to sixteen specific causes, including all cardiovascular diseases (ICD-10 codes: I00-I99, G45, G46), ischemic heart disease (I20-I25), heart failure (I50), heart rhythm disturbances (I47-I49), cerebrovascular diseases (I60-I69, G45, G46), stroke (I60, I61, I63, I64, G45, H34.1), peripheral vascular disease (I70-I89), all respiratory diseases (J00-J99), chronic obstructive pulmonary disease (COPD, J40-J44), asthma (J45, J46), pneumonia (J12-J18), diabetes (E10-E14), renal diseases (N00-N19), mental health conditions (F00-F99), neoplasms (C00-C97, D00-D48), and heat illness (T67, X30, E86). These specific causes were chosen based on previous reviews on the morbidity impacts of high temperature[2]

**Exposure measurements**

We have obtained the daily minimum and maximum temperatures from a national meteorological dataset (0.25° × 0.25° resolution) developed by Xavier [3]. We will use the weather of city centre to represent each city as we did in previous study [1]. In this study, daily mean temperature (the average of daily minimum and maximum temperatures) during hot seasons will be used to represent heat exposure [1]. Hot season will be defined as the city-specific four adjacent hottest months during 2000-2015.

**Socioeconomic and demographic data**

We have collected city-level socioeconomic and demographic data from the website of Brazilian Institute of Geography and Statistics (BIGS) (<https://www.ibge.gov.br/en/home-eng.html>). City-level literacy rate, urbanization rate, and population size came from the Brazilian Census 2000 and 2010, the data gaps for other years during 2000-2015 will be filled by linear interpolation. City-level household income from Brazil Census 2000 and 2010 are not comparable due to different statistical criteria. We will use the 2010 value which tends to be a better surrogate of the 16-year average than the 2000 value. Annul city-level GDP per capita during 2000-2015 were reported by BIGS. We have adjusted all GDP per capita and household income data to United States dollars (USD) at 2015 price, according to the consumer price index during 2001-2015 and the average exchange rate in 2015. From the Brazilian Census 2010, we have also collected the percentage of young population (0-19 years) and elderly population (60 years or above), as an indicator of population age structure.

City-level socioeconomic levels will be represented by 16-year (2000-2015) average literacy rate of people aged 15 years or above, 16-year average urbanization rate (the proportion of urban people), average monthly household income per capita in 2010, and 16-year average gross domestic product (GDP) per capita.

To make the results easy to understand, we will stratify the 1,814 cities into four groups (Q1~Q4) according to the quartiles (from the lowest to the highest quartile) of each socioeconomic indicator. We will also try to classify the cities into low income(LM, GDP per capita: less than 978 USD) ,lower middle income (LMI, 1,146-4,035 USD), upper middle income (UMI, 4,036-12,475 USD) and high-income (HI, >12,475 USD) according to World Bank’s 2015 standard.

**Statistical Analyses**

**1. Descriptive analyses**

Basic characteristics of hospitalizations, population structure, socioeconomic indicators, tempertures in Brazil during the study period will be summarized. Such as the number of all-cause and cause-specific hospitalizations, the sex and age distributions, and the mean/median tempertures and socioeconomic indicators during the study period. We will also use several maps to describe the geographical distribution of the temperature and socioeconomic indicators.

**2. Heat-hospitalization associations and the socioeconomic disparity**

We will use two-stage analyses to quantify the associations between heat exposure and risk of all-cause or cause-specific hospitalization, and to evaluate the inter-city socioeconomic disparities in the associations.

**2.1 First-stage analyses: modelling city-specific heat-hospitalization association**

We will use a time-stratified case-crossover design to evaluated the heat-hospitalization association for each city. Quasi-Poisson regression with a distributed lag model was used to estimate the city-specific association with equation below[1]:

$\mathrm{Log}\left( Y_{it} \right)=\alpha+cb\left( \mathrm{Temp}_{it} \right)+\beta\mathrm{Strata}_{it}+ \gamma\mathrm{DOW}_{it}+\delta\mathrm{Holiday}_{it}+\varepsilon_{it}$ (1)

where $Y_{it}$ represents the daily number of hospitalizations in city i on day t; $\alpha$ is the intercept; $\beta$, $\gamma$, and $\delta$ are the coefficients; $\varepsilon_{it}$ is the residual error. $\mathrm{Strata}_{it}$ is a stratum variable by combining year and calendar month to adjust for long-term trend and inter-month seasonal variations. $\mathrm{DOW}_{it}$ is a categorical variable to adjust for day of week. $\mathrm{Holiday}_{it}$ is a binary variable (being public holiday or not) to adjust for potential impacts of public holidays. $\mathrm{cb}\left( \mathrm{Temp}_{it} \right)$ is a two-dimensional (exposure-response dimension and lag-response dimension) cross-basis function to model the lagged effects of daily mean temperature. In each dimension, a specific smoothing function (e.g., linear function, nature cubic spline) will be used to define the shape of relationship[4, 5]. According to our preliminary analyses described before[1], we will use a linear function for the temperature-response dimension, and a natural cubic spline with three degrees of freedom (df) for lag-response dimension along 0−7 lag days.

In consistent with our previous study[1], the heat-hospitalization association will be reported as the cumulative relative risk (RR) of hospitalization [with 95% confidence intervals (CIs)] over lag 0−7 days associated with every 5℃ increase in daily mean temperature during hot seasons. The increased hospitalization risk associated with every 5℃ increase in daily mean temperature will be calculated as 100%×(RR-1).

**2.2 Second-stage analyses: evaluating pooled association and inter-city socioeconomic disparity in associations**

At the second stage, we will pool the city-specific estimates for all cities or cities at different socioeconomic groups (Q1~Q4 of each socioeconomic indicator, or classification according to World Bank), using a random-effect meta-analysis with maximum likelihood estimation.[6] This will yield a pooled estimation of the heat-hospitalization association at national level or within different socioeconomic strata. Using 1,814 city-specific effect estimates, we will use random effect meta-regression with the socioeconomic strata variable (e.g., urbanization quantiles) as the only meta-predictor to test whether the socioeconomic disparity in heat-hospitalisation was statistically significant between different socioeconomic groups.

**2.3 Subgroup analyses**

We will repeat above two-stage analyses based on hospitalizations of different subgroups, including:

- sex (male and female)
- age groups (0−19, 20−39, 40−59, and ≥60 years)
- sixteen specific causes of hospitalizations

**2.4 Sensitivity analyses**

We will perform a sensitivity analysis to test whether the socioeconomic disparity in heat vulnerability remained significant after adjusting for potential confounders in the meta-regression. For each socioeconomic indicator in the random effect meta-regression model, we will adjust for city-specific mean temperature, temperature range (maximum temperature minus minimum temperature), and the ratio of young population (0-19 years) and elderly population (60 years or above) as the indicator of population structure.

**4. Statistical software**

The maps will be draw with ArcGis desktop (version 10.6). All other analyses will be performed in R software (version 3.5.1), with several important R packages including “dplyr”(data cleaning),“dlnm”(distribute lag model at first stage), “mvmeta”(meta-analyses and meta-regression), “splines” (spline functions to model non-linear relationship) , “ggplot2”(visulization), “ggsci”(visulization).

**Modification based on the comments from editors and reviewers:**

According to the suggestion of reviewers, we made three revisions:

1. In the meta-regression, we replaced the socioeconomic quartiles with the continuous socioeconomic variable, because the reviewer suggested that it was inappropriate to categorize the independent variables in meta-regression. We also tried to add each socioeconomic variable to the meta-regression model as a natural cubic spline function with two to four degrees of freedom, in order to capture the potential non-linearity. However, the Bayesian information criterion (BIC) values of non-linear models were generally larger than or close to the BIC values of linear meta-regression model. This suggested that linear model outperformed non-linear models in the meta-regression, thus we kept using linear meta-regression model throughout the analyses.
2. We added the overall effect estimates (i.e., not stratified by socioeconomic levels) of the associations between heat exposure and sex-, age- and cause-specific hospitalizations.
3. We repeated our main analyses based on 1,723 cities with relatively small population size (ranging from 8,014 to 243,270), after excluding cities with population size larger than the 95th percentile of 1,814 cities. The city-level socioeconomic indicators in those small cities tend to be more accutate estimates of the residents’ true socioeconomic situations compared to those of large cities.

**References**

1. Zhao Q, Li S, Coelho M, Saldiva PHN, Hu K, Arblaster JM, et al. Geographic, Demographic, and Temporal Variations in the Association between Heat Exposure and Hospitalization in Brazil: A Nationwide Study between 2000 and 2015. Environ Health Persp. 2019;127(1):17001. https://doi.org/10.1289/ehp3889. PMID: 30620212.

2. Song X, Wang S, Hu Y, Yue M, Zhang T, Liu Y, et al. Impact of ambient temperature on morbidity and mortality: an overview of reviews. Sci Total Environ. 2017;586:241-54. https://doi.org/10.1016/j.scitotenv.2017.01.212. PMID: 28187945.

3. Xavier AC, King CW, Scanlon BR. Daily gridded meteorological variables in Brazil (1980-2013). Int J Climatol. 2016;36(6):2644-59. https://doi.org/10.1002/joc.4518.

4. Gasparrini A. Distributed Lag Linear and Non-Linear Models in R: The Package dlnm. J Stat Softw. 2011;43(8):1-20. PMID: 22003319.

5. Guo Y. Hourly associations between heat and ambulance calls. Environ Pollut. 2017;220:1424-8. https://doi.org/10.1016/j.envpol.2016.10.091. PMID: 27825842.

6. Gasparrini A, Armstrong B. Reducing and meta-analysing estimates from distributed lag non-linear models. Bmc Med Res Methodol. 2013;13. https://doi.org/10.1186/1471-2288-13-1. PMID: 23297754.
