# Supplementary material for: Socioeconomic level and associations between heat exposure and all-cause and cause-specific hospitalization in 1,814 Brazilian cities: A nationwide case-crossover study
Source: PLoS Med. 2020 Oct 8;17(10):e1003369. doi: 10.1371/journal.pmed.1003369 (PMC7544074; doi:10.1371/journal.pmed.1003369)
Supplement: S2 Text — (DOCX) [file pmed.1003369.s003.docx]

**Supplementary tables and figures**

Socioeconomic level and associations between heat exposure and all-cause and cause-specific hospitalization in 1,814 Brazilian cities: a nationwide case-crossover study

**Table of Contents**

1. **Fig A**. The distribution of the 16-year average population size for 1,814 cities
2. **Table A.** The case number and age distribution of all-cause and cause-specific hospitalization.
3. **Table B.** The Bayesian information criterion (BIC) values of linear and non-linear meta-regression models
4. **Table C.** The Pearson correlation matrix of city-level climatic, socioeconomic and demographic indicators for 1,814 cities in Brazil
5. **Fig B.** Mapping the average daily mean temperature during 2000-2015 hot seasons in 1,814 Brazilian cities.
6. **Fig C.** The relationship between city-level socioeconomic factors and the magnitude of heat-hospitalization association among 1,814 cities.
7. **Fig D.** The association between heat exposure (every 5℃ increase in daily mean temperature) and cause-specific hospitalization, stratified by literacy rate quartiles, and major causes.
8. **Fig E.** The association between heat exposure (every 5℃ increase in daily mean temperature) and cause-specific hospitalization, stratified by urbanization rate quartiles, and major causes.
9. **Fig F.** The association between heat exposure (every 5℃ increase in daily mean temperature) and cause-specific hospitalization, stratified by average household income quartiles, and major causes.
10. **Table D.** The association between heat exposure (every 5℃ increase in daily mean temperature) and cause-specific hospitalization, disparity between different GDP per capita classifications, by sex, age, and 16 specific causes.
11. **Table E.** The association between heat exposure (every 5℃ increase in daily mean temperature) and cause-specific hospitalization, disparity between different literacy rate quartiles, by sex, age, and 16 specific causes.
12. **Table F.** The association between heat exposure (every 5℃ increase in daily mean temperature) and cause-specific hospitalization, disparity between different urbanization rate quartiles, by sex, age, and 16 specific causes.
13. **Table G.** The association between heat exposure (every 5℃ increase in daily mean temperature) and cause-specific hospitalization, disparity between different household income quartiles, by sex, age, and 16 specific causes.
14. **Table H.** The association between heat exposure (every 5℃ increase in daily mean temperature) and cause-specific hospitalization, disparity between different GDP per capita quartiles, by sex, age, and 16 specific causes.
15. **Table I**. The number of hospitalization due to specific causes during 2000-2015 hot seasons among elderly people(≥60 years).
16. **Fig G.** The association between heat exposure (every 5℃ increase in daily mean temperature) and all-cause hospitalization, after excluding cities with population size larger than the 95th percentile of 1,814 cities.
17. **Fig H.** The relationship between city-level socioeconomic factors and the magnitude of heat-hospitalization association among 1,723 cities, after excluding cities with population size larger than the 95th percentile of 1,814 cities.
18. **Fig I.** The association between heat exposure (every 5℃ increase in daily mean temperature) and hospitalization due to different types of stroke during lag 0-7 days, stratified by GDP per capita classifications.

**
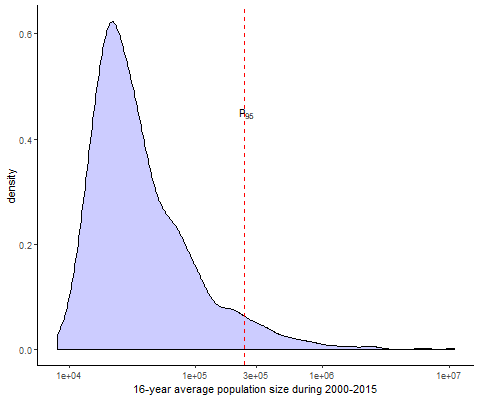
**

**Fig A. The distribution of the 16-year average population size for 1,814 cities**

Notes: P_95_ represents the 95th percentile, which corresponded to 243,271 in this figure. The x-axis is on log scale (1e+04 = 10,000; 1e+05 = 100,000 etc).

| **Table A.** The case number and age distribution of all-cause and cause-specific hospitalization. | | | | | | | |
| --- | --- | --- | --- | --- | --- | --- | --- |
| **Diseases** |  | **No. of cases by age groups, n (%)** | | | | | |
|  | **ICD-10 codes** | **0-19 years** | **20-39 years** | **40-59 years** | **≥60 years** | **age missing** | **Total** |
| All cardiovascular diseases | I00-I99, G45, G46 | 104,989(2.1) | 511,142(10.3) | 1,606,007(32.4) | 2,742,120(55.2) | 43(0.0) | 4,964,301(100.0) |
| Ischemic heart disease | I20-I25 | 2,420(0.3) | 37,063(4.0) | 373,131(40.3) | 514,027(55.5) | 6(0.0) | 926,647(100.0) |
| Heart failure | I50 | 19,286(1.7) | 56,623(4.9) | 287,874(25.0) | 786,022(68.4) | 1(0.0) | 1,149,806(100.0) |
| Heart rhythm disturbances | I47-I49 | 5,731(3.9) | 15,716(10.6) | 42,242(28.6) | 83,936(56.9) | 1(0.0) | 147,626(100.0) |
| Cerebrovascular diseases | I60-I69, G45,G46 | 12,070(1.3) | 57,554(6.0) | 269,264(27.9) | 626,347(64.9) | 5(0.0) | 965,240(100.0) |
| Stroke | I60, I61, I63, I64,G45, H34.1 | 7,471(1.0) | 43,571(5.6) | 213,982(27.3) | 519,027(66.2) | 3(0.0) | 784,054(100.0) |
| Peripheral vascular disease | I70-I89 | 29,566(3.1) | 240,032(25.2) | 372,564(39.1) | 310,999(32.6) | 25(0.0) | 953,186(100.0) |
| All respiratory diseases | J00-J99 | 2,455,897(47.8) | 558,904(10.9) | 700,905(13.7) | 1,418,451(27.6) | 20(0.0) | 5,134,177(100.0) |
| COPD | J40-J44 | 28,130(4.7) | 22,729(3.8) | 131,029(22.1) | 411,886(69.4) | 0(0.0) | 593,774(100.0) |
| Asthma | J45,J46 | 510,007(63.5) | 94,924(11.8) | 92,969(11.6) | 104,826(13.1) | 1(0.0) | 802,727(100.0) |
| Pneumonia | J12-J18 | 1,258,491(51.7) | 248,046(10.2) | 291,466(12.0) | 634,081(26.1) | 1(0.0) | 2,432,085(100.0) |
| Diabetes | E10-E14 | 32,706(5.9) | 58,386(10.6) | 179,515(32.4) | 282,743(51.1) | 1(0.0) | 553,351(100.0) |
| Renal diseases | N00-N19 | 207,362(22.4) | 250,411(27.0) | 219,332(23.7) | 249,654(26.9) | 4(0.0) | 926,763(100.0) |
| Mental health conditions | F00-F99 | 100,822(2.6) | 1,418,364(37.2) | 1,695,665(44.5) | 576,516(15.1) | 17194(0.5) | 3,808,561(100.0) |
| Neoplasms | C00-C97, D00-D48 | 257,151(10.3) | 446,223(17.8) | 950,954(38.0) | 845,710(33.8) | 90(0.0) | 2,500,128(100.0) |
| Heat illness | T67, X30, E86 | 130,754(50.5) | 27,872(10.8) | 32,176(12.4) | 68,053(26.3) | 0(0.0) | 258,855(100.0) |
| **All-cause** | — | 12,290,723(25.0) | 16,457,693(33.5) | 10,310,760(21.0) | 9,367,904(19.1) | 718,917(1.5) | 49,145,997(100.0) |
| Note: COPD, Chronic Obstructive Pulmonary Disease. ICD, International Classification of Diseases. | | | | | | | |

| **Table B.** The Bayesian information criterion (BIC) values of linear and non-linear meta-regression models | | | | |
| --- | --- | --- | --- | --- |
| Meta-predictors | Linear model | Non-linear model | | |
|  |  | ns, df=2 | ns, df=3 | ns, df=4 |
| Literacy rate | -4051.8 | -4047.6 | -4052.8 | -4049.3 |
| Urbanization rate | -4051.2 | -4043.8 | -4036.4 | -4029.2 |
| Average household income | -4052.5 | -4045.7 | -4040.3 | -4033.2 |
| GDP per capita | -4054.8 | -4048.0 | -4040.7 | -4033.8 |

Notes: ns, natural cubic spline; df, degree of freedom. GDP, gross domestic product.

| **Table C.** The Pearson correlation matrix of city-level climatic, socioeconomic and demographic indicators for 1,814 cities in Brazil | | | | | | | | | |
| --- | --- | --- | --- | --- | --- | --- | --- | --- | --- |
|  | Literacy rate | Urbanization rate | Average household income | GDP per capita | Percentage of population aged 0~19 | Percentage of population aged 60 or above | The ratio of elderly and young population | Mean temperature |  |
| Literacy rate | 1.00 |  |  |  |  |  |  |  |  |
| Urbanization rate | 0.70*** | 1.00 |  |  |  |  |  |  |  |
| Average household income | 0.84*** | 0.69*** | 1.00 |  |  |  |  |  |  |
| GDP per capita | 0.51*** | 0.40*** | 0.57*** | 1.00 |  |  |  |  |  |
| Percentage of population aged 0~19 | -0.73*** | -0.58*** | -0.78*** | -0.39*** | 1.00 |  |  |  |  |
| Percentage of population aged 60 or above | 0.08*** | -0.02 | 0.18*** | -0.06** | -0.59*** | 1.00 |  |  |  |
| The ratio of elderly and young population# | 0.37*** | 0.23*** | 0.46*** | 0.12*** | -0.81*** | 0.93*** | 1.00 |  |  |
| Mean temperature | -0.63*** | -0.32*** | -0.53*** | -0.31*** | 0.52*** | -0.20*** | -0.33*** | 1.00 |  |
| Temperature range | 0.70*** | 0.34*** | 0.64*** | 0.36*** | -0.54*** | 0.21*** | 0.37*** | -0.63*** |  |
| *P<0.05; **P<0.01; ****P*<0.001; ^#^ the ratio of elderly population (≥60 years) and young population (0-19 years). GDP, gross domestic product. | | | | | | | | |  |


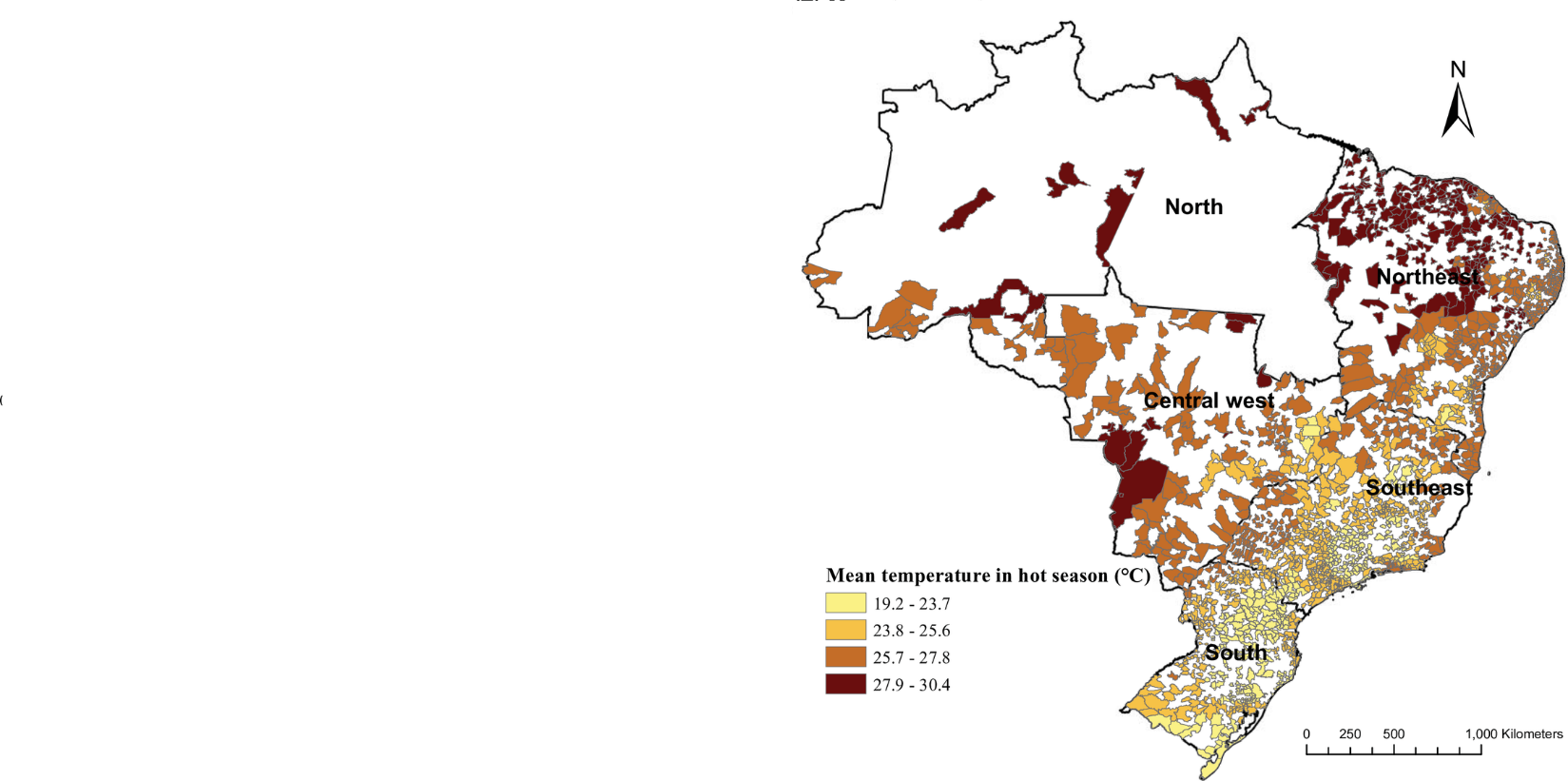


**Fig B.** Mapping the average daily mean temperature during 2000-2015 hot seasons in 1,814 Brazilian cities.

Note: Hot season was defined as city-specific four adjacent hottest months during 2000-2015. The base map of this figure was downloaded from the Brazilian Institute of Geography and Statistics (https://www.ibge.gov.br/); the base map was free and open-access.


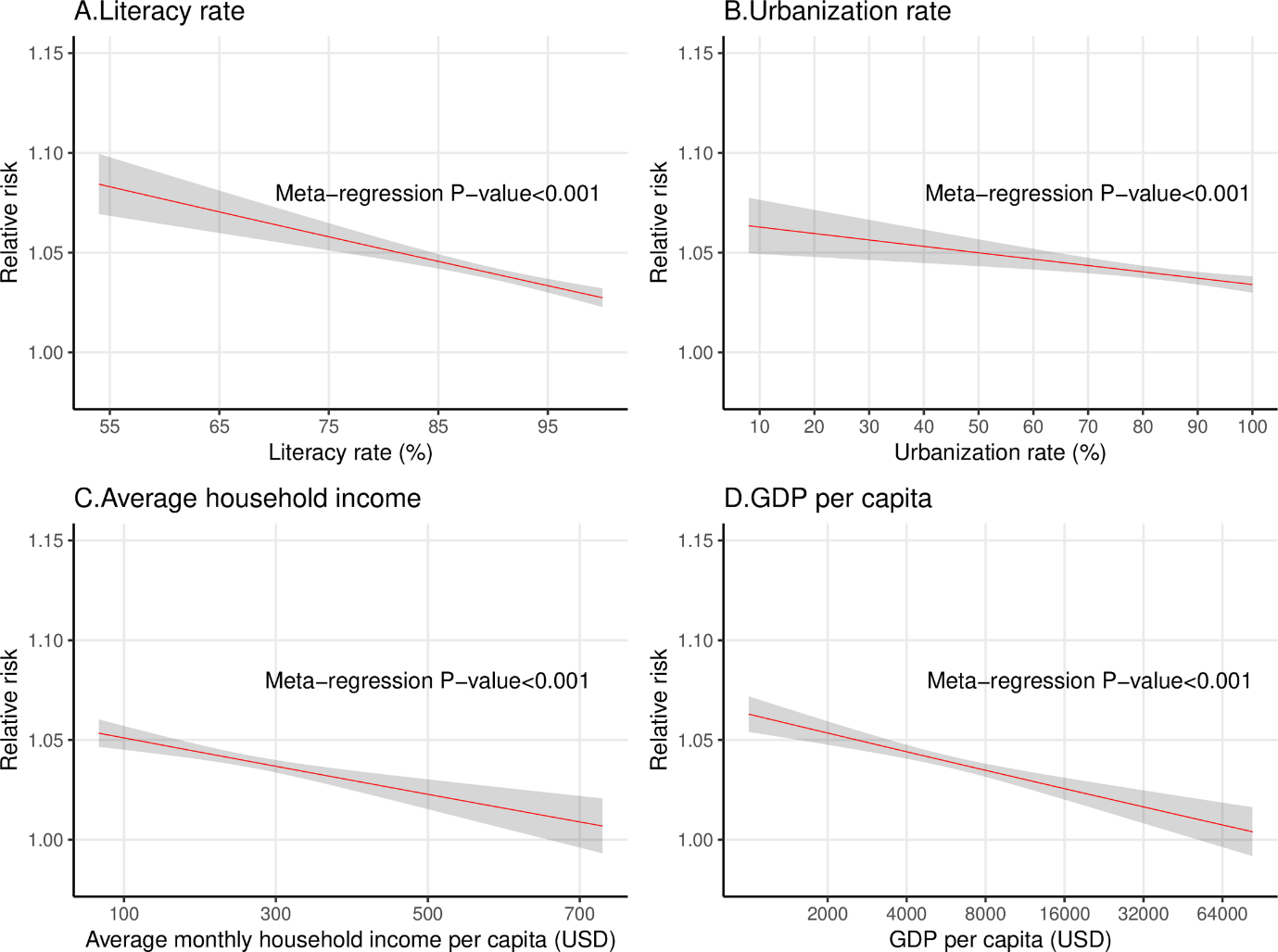
 **Fig C.** The relationship between city-level socioeconomic factors and the magnitude of heat-hospitalization association among 1,814 cities.

Note: relative risk (RR) represents the association between heat exposure (every 5℃ increase in daily mean temperature) and hospitalization during lag 0-7 days. The shadowed area represents 95% confidence intervals. GDP, gross domestic product. USD, United States Dollar. The relationship between city-specific RR and four socioeconomic indicators were fitted separately by meta-regression, adjusting for no other covariates. The x-axis of Fig D was is in log scale, because we added log (GDP per capita) rather than GDP per capita to the meta-regression model.


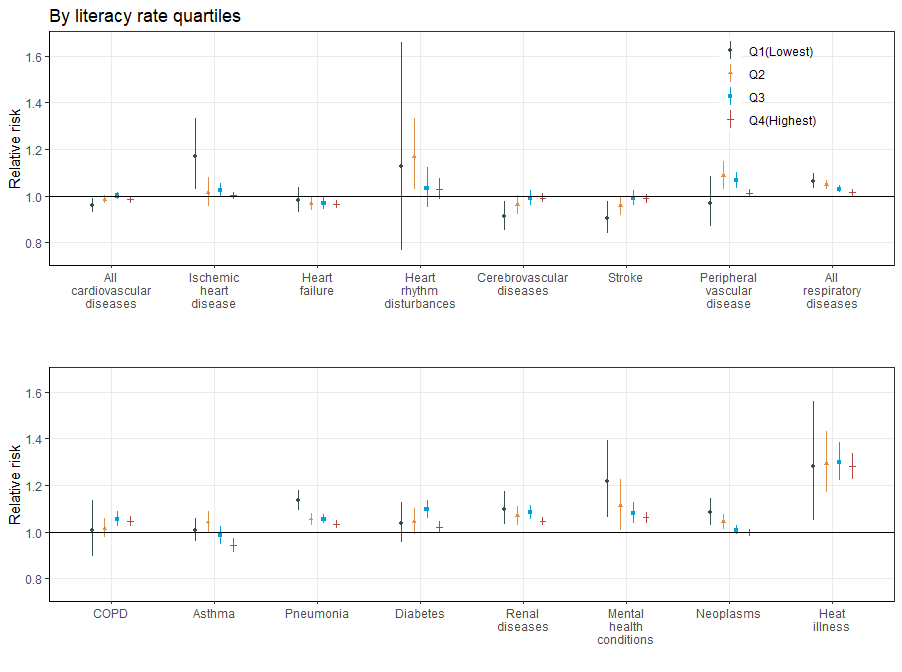


**Fig D.** The association between heat exposure (every 5℃ increase in daily mean temperature) and cause-specific hospitalization, stratified by literacy rate quartiles, and major causes.

Notes: COPD, Chronic Obstructive Pulmonary Disease. Q1-Q4 represented four quartiles.


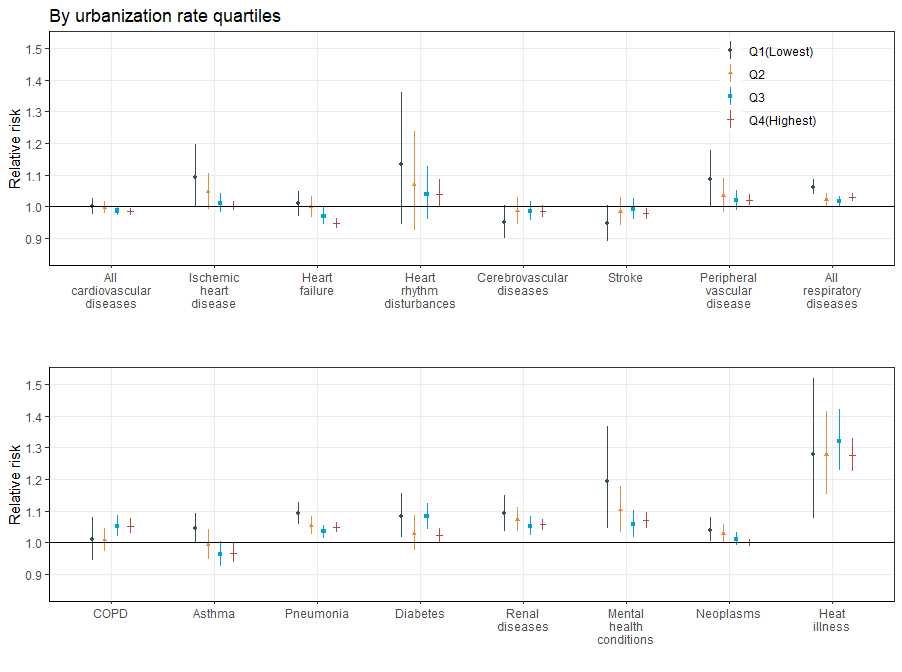


**Fig E.** The association between heat exposure (every 5℃ increase in daily mean temperature) and cause-specific hospitalization, stratified by urbanization rate quartiles, and major causes.

Notes: COPD, Chronic Obstructive Pulmonary Disease. Q1-Q4 represented four quartiles.


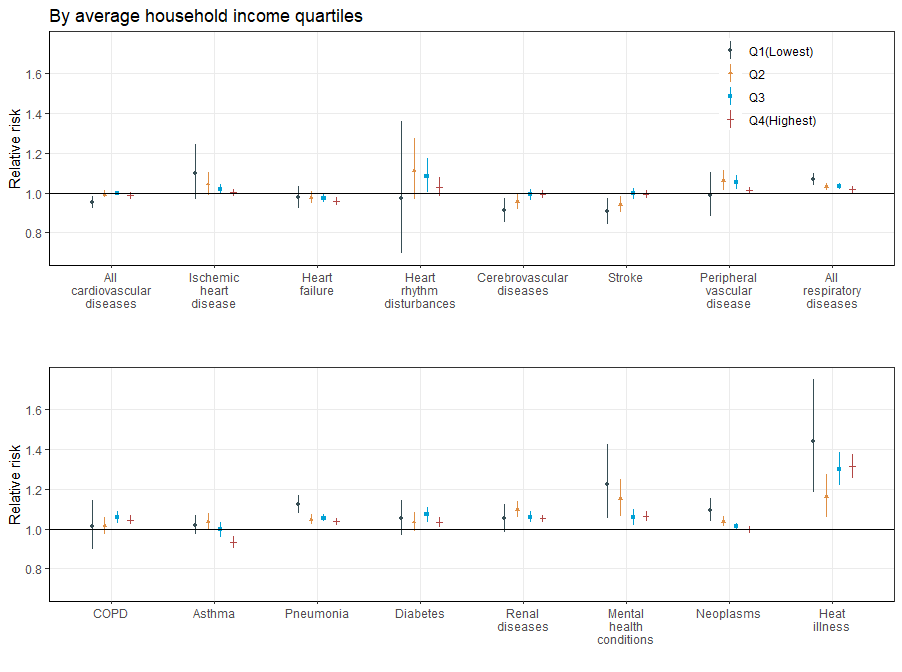


**Fig F.** The association between heat exposure (every 5℃ increase in daily mean temperature) and cause-specific hospitalization, stratified by average household income quartiles, and major causes.

Notes: COPD, Chronic Obstructive Pulmonary Disease. Q1-Q4 represented four quartiles.

| **Table D.** The association between heat exposure (every 5℃ increase in daily mean temperature) and cause-specific hospitalization, disparity between different GDP per capita classifications, by sex, age, and 16 specific causes. | | | | | | | |
| --- | --- | --- | --- | --- | --- | --- | --- |
|  | All cities combined, RR (95%CI) | GDP per capita classification, RR (95%CI) | | |  | Meta-regression | |
| **Subgroup** |  | Lower middle income | Upper middle income | High income |  | Coefficient | P-value |
| Female, all-cause | 1.037(1.034, 1.041) | 1.051(1.043, 1.058) | 1.034(1.030, 1.039) | 1.022(1.014, 1.031) |  | -1.5E-02 | <0.001 |
| Male, all-cause | 1.041(1.037, 1.045) | 1.050(1.042, 1.059) | 1.040(1.034, 1.045) | 1.030(1.019, 1.042) |  | -1.5E-02 | <0.001 |
| 0-19 years, all-cause | 1.069(1.064, 1.075) | 1.099(1.087, 1.111) | 1.061(1.055, 1.068) | 1.052(1.041, 1.063) |  | -3.2E-02 | <0.001 |
| 20-39 years, all-cause | 1.040(1.036, 1.045) | 1.048(1.039, 1.057) | 1.040(1.035, 1.045) | 1.029(1.020, 1.038) |  | -1.1E-02 | 0.002 |
| 40-59 years, all-cause | 1.019(1.014, 1.025) | 1.039(1.026, 1.052) | 1.016(1.010, 1.023) | 0.999(0.981, 1.018) |  | -2.0E-02 | <0.001 |
| 60 years or above, all-cause | 1.019(1.014, 1.024) | 0.997(0.985, 1.009) | 1.025(1.019, 1.031) | 1.020(1.006, 1.035) |  | 1.2E-02 | 0.011 |
| **Cause-specific** |  |  |  |  |  |  |  |
| All cardiovascular diseases | 0.989(0.982, 0.995) | 0.977(0.962, 0.991) | 0.992(0.985, 1.000) | 0.987(0.972, 1.002) |  | 2.4E-03 | 0.699 |
| Ischemic heart disease | 1.013(0.999, 1.026) | 1.056(0.998, 1.118) | 1.006(0.991, 1.021) | 1.005(0.979, 1.031) |  | -3.0E-02 | 0.029 |
| Heart failure | 0.968(0.955, 0.980) | 0.964(0.937, 0.993) | 0.970(0.955, 0.984) | 0.963(0.921, 1.008) |  | -1.3E-02 | 0.275 |
| Heart rhythm disturbances | 1.072(1.015, 1.131) | 1.115(0.983, 1.265) | 1.047(0.998, 1.098) | 1.009(0.889, 1.145) |  | -4.7E-02 | 0.209 |
| Cerebrovascular diseases | 0.984(0.970, 0.998) | 0.941(0.910, 0.973) | 0.996(0.978, 1.013) | 0.984(0.953, 1.016) |  | 3.3E-02 | 0.019 |
| Stroke | 0.981(0.966, 0.996) | 0.930(0.898, 0.962) | 0.996(0.977, 1.014) | 0.983(0.954, 1.014) |  | 3.5E-02 | 0.015 |
| Peripheral vascular disease | 1.030(1.015, 1.045) | 1.035(0.987, 1.085) | 1.033(1.016, 1.050) | 1.002(0.969, 1.036) |  | -1.3E-02 | 0.374 |
| All respiratory diseases | 1.030(1.022, 1.037) | 1.055(1.038, 1.073) | 1.021(1.012, 1.030) | 1.025(1.006, 1.045) |  | -2.5E-02 | <0.001 |
| COPD | 1.040(1.023, 1.057) | 1.016(0.965, 1.069) | 1.040(1.022, 1.059) | 1.047(1.004, 1.093) |  | 1.7E-02 | 0.305 |
| Asthma | 0.987(0.967, 1.007) | 1.037(1.003, 1.071) | 0.963(0.938, 0.989) | 0.936(0.879, 0.997) |  | -6.3E-02 | <0.001 |
| Pneumonia | 1.052(1.041, 1.062) | 1.080(1.056, 1.104) | 1.044(1.031, 1.057) | 1.038(1.011, 1.065) |  | -3.1E-02 | 0.001 |
| Diabetes | 1.046(1.027, 1.065) | 1.038(0.996, 1.083) | 1.044(1.021, 1.067) | 1.054(1.001, 1.110) |  | 2.6E-03 | 0.878 |
| Renal diseases | 1.062(1.048, 1.075) | 1.096(1.062, 1.131) | 1.052(1.036, 1.069) | 1.049(1.018, 1.080) |  | -2.2E-02 | 0.073 |
| Mental health conditions | 1.085(1.059, 1.111) | 1.172(1.084, 1.268) | 1.065(1.040, 1.090) | 1.055(0.986, 1.130) |  | -4.8E-02 | 0.034 |
| Neoplasms | 1.008(0.999, 1.016) | 1.031(1.007, 1.055) | 1.006(0.997, 1.016) | 0.999(0.979, 1.020) |  | -2.8E-02 | 0.001 |
| Heat illness | 1.303(1.244, 1.366) | 1.278(1.161, 1.406) | 1.307(1.255, 1.362) | 1.154(1.014, 1.313) |  | -9.5E-04 | 0.980 |
| Note: RR, relative risk; CI, confidence interval; COPD, Chronic Obstructive Pulmonary Disease. In the meta-regression, we modelled the 1,814 city-specific effect estimates against the city-level log(GDP per capita). The coefficient represented the change in log(RR) associated with an inter-quartile increase in log(GDP per capita). | | | | | | | |

| **Table E.** The association between heat exposure (every 5℃ increase in daily mean temperature) and cause-specific hospitalization, disparity between different literacy rate quartiles, by sex, age, and 16 specific causes. | | | | | | | | | | | | | | | |
| --- | --- | --- | --- | --- | --- | --- | --- | --- | --- | --- | --- | --- | --- | --- | --- |
|  | | Literacy rate (%) quartiles, RR (95%CI) | | | | | | | | |  | Meta-regression | | | |
| **Subgroup** | | Q1(54.6,75.5] | | Q2(75.5,89.1] | | Q3(89.1,93.3] | | Q4(93.3,98.3] | | |  | Coefficient | | P-value | |
| Female, all-cause | | 1.065(1.053, 1.077) | | 1.045(1.035, 1.054) | | 1.044(1.036, 1.051) | | 1.027(1.023, 1.031) | | |  | -2.3E-02 | | <0.001 | |
| Male, all-cause | | 1.070(1.051, 1.088) | | 1.045(1.034, 1.056) | | 1.047(1.039, 1.055) | | 1.033(1.028, 1.039) | | |  | -2.1E-02 | | <0.001 | |
| 0-19 years, all-cause | | 1.137(1.116, 1.159) | | 1.087(1.073, 1.102) | | 1.070(1.059, 1.081) | | 1.052(1.045, 1.059) | | |  | -5.4E-02 | | <0.001 | |
| 20-39 years, all-cause | | 1.061(1.045, 1.078) | | 1.043(1.031, 1.056) | | 1.048(1.038, 1.057) | | 1.034(1.029, 1.039) | | |  | -1.5E-02 | | 0.003 | |
| 40-59 years, all-cause | | 1.045(1.022, 1.070) | | 1.029(1.013, 1.045) | | 1.027(1.015, 1.038) | | 1.011(1.004, 1.018) | | |  | -2.4E-02 | | 0.001 | |
| 60 years or above, all-cause | | 0.983(0.961, 1.005) | | 1.009(0.995, 1.024) | | 1.034(1.025, 1.044) | | 1.017(1.011, 1.024) | | |  | 2.5E-02 | | <0.001 | |
| **Cause-specific** | |  | |  | |  | |  | | |  |  | |  | |
| All cardiovascular diseases | | 0.961(0.932, 0.992) | | 0.986(0.968, 1.004) | | 1.001(0.987, 1.015) | | 0.986(0.978, 0.994) | | |  | 1.4E-02 | | 0.154 | |
| Ischemic heart disease | | 1.172(1.029, 1.335) | | 1.015(0.954, 1.080) | | 1.025(0.996, 1.055) | | 1.002(0.987, 1.017) | | |  | -5.8E-02 | | 0.011 | |
| Heart failure | | 0.982(0.929, 1.038) | | 0.968(0.937, 1.001) | | 0.968(0.943, 0.992) | | 0.966(0.949, 0.983) | | |  | -1.4E-02 | | 0.404 | |
| Heart rhythm disturbances | | 1.129(0.769, 1.658) | | 1.171(1.027, 1.334) | | 1.034(0.953, 1.122) | | 1.030(0.985, 1.076) | | |  | -2.5E-02 | | 0.425 | |
| Cerebrovascular diseases | | 0.914(0.855, 0.978) | | 0.963(0.923, 1.004) | | 0.992(0.962, 1.023) | | 0.991(0.973, 1.010) | | |  | 6.3E-02 | | 0.002 | |
| Stroke | | 0.906(0.841, 0.976) | | 0.957(0.916, 1.000) | | 0.991(0.961, 1.023) | | 0.989(0.970, 1.008) | | |  | 6.6E-02 | | 0.001 | |
| Peripheral vascular disease | | 0.971(0.870, 1.083) | | 1.086(1.027, 1.149) | | 1.067(1.031, 1.103) | | 1.011(0.995, 1.027) | | |  | -1.8E-02 | | 0.421 | |
| All respiratory diseases | | 1.064(1.033, 1.097) | | 1.047(1.028, 1.067) | | 1.030(1.016, 1.044) | | 1.017(1.007, 1.027) | | |  | -3.2E-02 | | <0.001 | |
| COPD | | 1.009(0.896, 1.135) | | 1.016(0.976, 1.058) | | 1.055(1.024, 1.088) | | 1.046(1.026, 1.067) | | |  | 5.6E-02 | | 0.013 | |
| Asthma | | 1.008(0.960, 1.059) | | 1.042(0.997, 1.089) | | 0.984(0.948, 1.023) | | 0.943(0.913, 0.974) | | |  | -5.8E-02 | | 0.004 | |
| Pneumonia | | 1.136(1.093, 1.180) | | 1.055(1.029, 1.082) | | 1.056(1.036, 1.077) | | 1.033(1.019, 1.048) | | |  | -5.1E-02 | | <0.001 | |
| Diabetes | | 1.037(0.954, 1.127) | | 1.044(0.989, 1.102) | | 1.095(1.057, 1.135) | | 1.022(1.000, 1.045) | | |  | -1.6E-02 | | 0.478 | |
| Renal diseases | | 1.099(1.031, 1.172) | | 1.070(1.029, 1.112) | | 1.084(1.054, 1.114) | | 1.045(1.029, 1.062) | | |  | -2.4E-02 | | 0.176 | |
| Mental health conditions | | 1.216(1.063, 1.392) | | 1.113(1.009, 1.227) | | 1.081(1.038, 1.126) | | 1.062(1.039, 1.086) | | |  | -6.4E-02 | | 0.029 | |
| Neoplasms | | 1.084(1.028, 1.143) | | 1.043(1.012, 1.074) | | 1.009(0.990, 1.028) | | 1.000(0.990, 1.010) | | |  | -6.1E-02 | | <0.001 | |
| Heat illness | | 1.280(1.051, 1.560) | | 1.294(1.170, 1.431) | | 1.299(1.220, 1.384) | | 1.281(1.227, 1.338) | | |  | -9.1E-05 | | 0.998 | |
| Note: RR, relative risk; CI, confidence interval; COPD, Chronic Obstructive Pulmonary Disease. In the meta-regression, we modelled the 1,814 city-specific effect estimates against the city-level literacy rate. The coefficient represented the change in log(RR) associated with an inter-quartile increase in literacy rate. | | | | | | | | | | | | | | | |
| **Table F.** The association between heat exposure (every 5℃ increase in daily mean temperature) and cause-specific hospitalization, disparity between different urbanization rate quartiles, by sex, age, and 16 specific causes. | | | | | | | | | | | | | | |  |
|  | Urbanization rate (%) quartiles | | | | | | | |  | Meta-regression | | | | |  |
| **Subgroup** | Q1(8.4,60.7] | | Q2(60.7,79.8] | | Q3(79.8,91.5] | | Q4(91.5,100] | |  | Coefficient | | | P-value | |  |
| Female, all-cause | 1.052(1.041, 1.063) | | 1.041(1.031, 1.050) | | 1.038(1.031, 1.045) | | 1.032(1.027, 1.037) | |  | -1.0E-02 | | | 0.001 | |  |
| Male, all-cause | 1.060(1.046, 1.074) | | 1.042(1.031, 1.053) | | 1.039(1.031, 1.048) | | 1.037(1.032, 1.043) | |  | -9.1E-03 | | | 0.018 | |  |
| 0-19 years, all-cause | 1.105(1.088, 1.123) | | 1.076(1.061, 1.091) | | 1.066(1.055, 1.078) | | 1.061(1.054, 1.068) | |  | -2.1E-02 | | | <0.001 | |  |
| 20-39 years, all-cause | 1.055(1.040, 1.070) | | 1.043(1.031, 1.056) | | 1.041(1.032, 1.050) | | 1.037(1.032, 1.042) | |  | -8.4E-03 | | | 0.033 | |  |
| 40-59 years, all-cause | 1.041(1.021, 1.061) | | 1.020(1.005, 1.034) | | 1.019(1.007, 1.031) | | 1.016(1.008, 1.023) | |  | -1.0E-02 | | | 0.057 | |  |
| 60 years or above, all-cause | 1.002(0.985, 1.019) | | 1.024(1.011, 1.037) | | 1.027(1.017, 1.036) | | 1.017(1.010, 1.024) | |  | 4.2E-03 | | | 0.382 | |  |
| **Cause-specific** |  | |  | |  | |  | |  |  | | |  | |  |
| All cardiovascular diseases | 1.002(0.977, 1.027) | | 0.998(0.980, 1.017) | | 0.987(0.974, 1.001) | | 0.985(0.977, 0.993) | |  | -1.4E-02 | | | 0.038 | |  |
| Ischemic heart disease | 1.093(0.998, 1.199) | | 1.047(0.992, 1.104) | | 1.011(0.981, 1.043) | | 1.003(0.988, 1.018) | |  | -3.6E-02 | | | 0.018 | |  |
| Heart failure | 1.010(0.971, 1.050) | | 1.000(0.966, 1.034) | | 0.970(0.944, 0.997) | | 0.947(0.931, 0.964) | |  | -4.3E-02 | | | <0.001 | |  |
| Heart rhythm disturbances | 1.133(0.943, 1.362) | | 1.070(0.927, 1.237) | | 1.041(0.961, 1.127) | | 1.041(0.998, 1.086) | |  | -4.2E-02 | | | 0.253 | |  |
| Cerebrovascular diseases | 0.950(0.899, 1.003) | | 0.986(0.945, 1.029) | | 0.986(0.956, 1.017) | | 0.986(0.968, 1.004) | |  | 1.2E-02 | | | 0.410 | |  |
| Stroke | 0.946(0.893, 1.004) | | 0.985(0.942, 1.030) | | 0.993(0.961, 1.026) | | 0.978(0.960, 0.997) | |  | 9.3E-03 | | | 0.541 | |  |
| Peripheral vascular disease | 1.087(1.002, 1.178) | | 1.036(0.983, 1.091) | | 1.020(0.990, 1.052) | | 1.021(1.004, 1.038) | |  | -3.4E-02 | | | 0.030 | |  |
| All respiratory diseases | 1.061(1.038, 1.086) | | 1.022(1.003, 1.042) | | 1.017(1.003, 1.032) | | 1.031(1.021, 1.041) | |  | -7.2E-03 | | | 0.275 | |  |
| COPD | 1.010(0.945, 1.079) | | 1.008(0.972, 1.047) | | 1.052(1.020, 1.086) | | 1.053(1.030, 1.077) | |  | 3.0E-02 | | | 0.045 | |  |
| Asthma | 1.046(1.002, 1.092) | | 0.994(0.948, 1.043) | | 0.964(0.926, 1.004) | | 0.966(0.937, 0.997) | |  | -4.5E-02 | | | 0.006 | |  |
| Pneumonia | 1.093(1.060, 1.127) | | 1.055(1.028, 1.082) | | 1.035(1.015, 1.056) | | 1.049(1.034, 1.064) | |  | -1.3E-02 | | | 0.171 | |  |
| Diabetes | 1.084(1.017, 1.156) | | 1.029(0.975, 1.086) | | 1.083(1.042, 1.125) | | 1.023(1.000, 1.046) | |  | -2.3E-02 | | | 0.179 | |  |
| Renal diseases | 1.092(1.037, 1.150) | | 1.073(1.036, 1.112) | | 1.053(1.024, 1.083) | | 1.057(1.039, 1.075) | |  | -1.0E-02 | | | 0.427 | |  |
| Mental health conditions | 1.195(1.045, 1.366) | | 1.103(1.032, 1.179) | | 1.059(1.018, 1.101) | | 1.071(1.045, 1.097) | |  | -3.8E-02 | | | 0.088 | |  |
| Neoplasms | 1.041(1.004, 1.079) | | 1.029(1.000, 1.058) | | 1.012(0.993, 1.032) | | 1.001(0.991, 1.011) | |  | -2.1E-02 | | | 0.034 | |  |
| Heat illness | 1.280(1.079, 1.518) | | 1.278(1.154, 1.415) | | 1.321(1.228, 1.421) | | 1.278(1.227, 1.331) | |  | -8.4E-03 | | | 0.827 | |  |
| Note: RR, relative risk; CI, confidence interval; COPD, Chronic Obstructive Pulmonary Disease. In the meta-regression, we modelled the 1,814 city-specific effect estimates against the city-level urbanization rate. The coefficient represented the change in log(RR) associated with an inter-quartile increase in urbanization rate. | | | | | | | | | | | | | | |  |
| **Table G.** The association between heat exposure (every 5℃ increase in daily mean temperature) and cause-specific hospitalization, disparity between different household income quartiles, by sex, age, and 16 specific causes. | | | | | | | | | | | | | | |  |
|  | Average household income (USD) quartiles | | | | | | | |  | Meta-regression | | | | |  |
| **Subgroup** | Q1(67,123] | | Q2(123,217] | | Q3(217,275] | | Q4(275,729] | |  | Coefficient | | | P-value | |  |
| Female, all-cause | 1.056(1.044, 1.069) | | 1.040(1.031, 1.048) | | 1.040(1.033, 1.047) | | 1.030(1.026, 1.035) | |  | -1.1E-02 | | | <0.001 | |  |
| Male, all-cause | 1.057(1.040, 1.075) | | 1.054(1.043, 1.064) | | 1.039(1.032, 1.047) | | 1.035(1.029, 1.041) | |  | -9.3E-03 | | | 0.002 | |  |
| 0-19 years, all-cause | 1.129(1.109, 1.149) | | 1.069(1.056, 1.083) | | 1.078(1.068, 1.089) | | 1.052(1.045, 1.059) | |  | -2.5E-02 | | | <0.001 | |  |
| 20-39 years, all-cause | 1.051(1.035, 1.068) | | 1.049(1.039, 1.060) | | 1.034(1.026, 1.042) | | 1.039(1.033, 1.045) | |  | -6.3E-03 | | | 0.022 | |  |
| 40-59 years, all-cause | 1.036(1.012, 1.060) | | 1.039(1.025, 1.052) | | 1.018(1.008, 1.029) | | 1.013(1.005, 1.020) | |  | -8.5E-03 | | | 0.043 | |  |
| 60 years or above, all-cause | 0.965(0.944, 0.987) | | 1.017(1.004, 1.029) | | 1.025(1.015, 1.034) | | 1.022(1.016, 1.029) | |  | 6.7E-03 | | | 0.063 | |  |
| **Cause-specific** |  | |  | |  | |  | |  |  | | |  | |  |
| All cardiovascular diseases | 0.951(0.921, 0.982) | | 0.993(0.975, 1.010) | | 0.997(0.985, 1.010) | | 0.986(0.978, 0.994) | |  | -1.9E-03 | | | 0.687 | |  |
| Ischemic heart disease | 1.096(0.965, 1.245) | | 1.044(0.987, 1.105) | | 1.018(0.992, 1.045) | | 1.000(0.985, 1.016) | |  | -2.4E-02 | | | 0.015 | |  |
| Heart failure | 0.977(0.924, 1.032) | | 0.976(0.946, 1.007) | | 0.974(0.952, 0.997) | | 0.959(0.941, 0.978) | |  | -1.6E-02 | | | 0.095 | |  |
| Heart rhythm disturbances | 0.972(0.695, 1.359) | | 1.112(0.969, 1.276) | | 1.085(1.005, 1.172) | | 1.030(0.982, 1.080) | |  | -3.6E-02 | | | 0.315 | |  |
| Cerebrovascular diseases | 0.909(0.852, 0.971) | | 0.958(0.919, 0.998) | | 0.991(0.963, 1.020) | | 0.994(0.976, 1.013) | |  | 2.6E-02 | | | 0.014 | |  |
| Stroke | 0.905(0.842, 0.972) | | 0.942(0.903, 0.983) | | 0.996(0.967, 1.025) | | 0.992(0.973, 1.011) | |  | 2.5E-02 | | | 0.022 | |  |
| Peripheral vascular disease | 0.986(0.881, 1.104) | | 1.062(1.013, 1.115) | | 1.053(1.020, 1.086) | | 1.013(0.996, 1.030) | |  | -2.3E-02 | | | 0.032 | |  |
| All respiratory diseases | 1.069(1.038, 1.100) | | 1.032(1.015, 1.050) | | 1.033(1.019, 1.047) | | 1.018(1.008, 1.029) | |  | -1.8E-02 | | | 0.002 | |  |
| COPD | 1.012(0.897, 1.142) | | 1.014(0.973, 1.056) | | 1.056(1.027, 1.086) | | 1.044(1.021, 1.067) | |  | 1.4E-02 | | | 0.285 | |  |
| Asthma | 1.019(0.971, 1.069) | | 1.035(0.991, 1.081) | | 0.996(0.959, 1.034) | | 0.931(0.901, 0.963) | |  | -6.2E-02 | | | <0.001 | |  |
| Pneumonia | 1.122(1.079, 1.167) | | 1.047(1.024, 1.071) | | 1.055(1.035, 1.075) | | 1.037(1.021, 1.053) | |  | -2.2E-02 | | | 0.005 | |  |
| Diabetes | 1.053(0.969, 1.145) | | 1.033(0.986, 1.083) | | 1.071(1.035, 1.109) | | 1.034(1.010, 1.058) | |  | -1.8E-03 | | | 0.897 | |  |
| Renal diseases | 1.051(0.984, 1.122) | | 1.097(1.058, 1.136) | | 1.060(1.033, 1.087) | | 1.051(1.034, 1.069) | |  | -1.5E-02 | | | 0.082 | |  |
| Mental health conditions | 1.225(1.053, 1.425) | | 1.152(1.064, 1.248) | | 1.057(1.016, 1.099) | | 1.062(1.038, 1.087) | |  | -3.6E-02 | | | 0.074 | |  |
| Neoplasms | 1.094(1.039, 1.151) | | 1.036(1.010, 1.063) | | 1.011(0.993, 1.029) | | 0.998(0.988, 1.008) | |  | -1.3E-02 | | | 0.018 | |  |
| Heat illness | 1.440(1.183, 1.753) | | 1.162(1.058, 1.277) | | 1.301(1.221, 1.386) | | 1.315(1.256, 1.376) | |  | 9.0E-03 | | | 0.812 | |  |
| Note: RR, relative risk; CI, confidence interval; COPD, Chronic Obstructive Pulmonary Disease. In the meta-regression, we modelled the 1,814 city-specific effect estimates against the city-level average household income. The coefficient represented the change in log(RR) associated with an inter-quartile increase in household income. | | | | | | | | | | | | | | |  |
| **Table H.** The association between heat exposure (every 5℃ increase in daily mean temperature) and cause-specific hospitalization, disparity between different GDP per capita quartiles, by sex, age, and 16 specific causes. | | | | | | | | | | | | | | |  |
|  | GDP per capita (USD) quartiles | | | | | | | |  | Meta-regression | | | | |  |
| **Subgroup** | Q1[978,2205] | | Q2(2205,4406] | | Q3(4406,6878] | | Q4(6878,83307] | |  | Coefficient | | | P-value | |  |
| Female, all-cause | 1.055(1.043, 1.068) | | 1.047(1.039, 1.056) | | 1.039(1.033, 1.045) | | 1.027(1.022, 1.032) | |  | -1.5E-02 | | | <0.001 | |  |
| Male, all-cause | 1.062(1.045, 1.079) | | 1.047(1.038, 1.056) | | 1.045(1.037, 1.053) | | 1.032(1.026, 1.038) | |  | -1.5E-02 | | | <0.001 | |  |
| 0-19 years, all-cause | 1.129(1.111, 1.149) | | 1.076(1.063, 1.090) | | 1.070(1.060, 1.080) | | 1.051(1.044, 1.059) | |  | -3.2E-02 | | | <0.001 | |  |
| 20-39 years, all-cause | 1.051(1.036, 1.067) | | 1.046(1.036, 1.056) | | 1.043(1.035, 1.052) | | 1.034(1.029, 1.040) | |  | -1.1E-02 | | | 0.002 | |  |
| 40-59 years, all-cause | 1.036(1.014, 1.058) | | 1.041(1.028, 1.055) | | 1.018(1.007, 1.028) | | 1.010(1.002, 1.017) | |  | -2.0E-02 | | | <0.001 | |  |
| 60 years or above, all-cause | 0.976(0.956, 0.995) | | 1.016(1.004, 1.028) | | 1.032(1.023, 1.042) | | 1.018(1.011, 1.024) | |  | 1.2E-02 | | | 0.011 | |  |
| **Cause-specific** |  | |  | |  | |  | |  |  | | |  | |  |
| All cardiovascular diseases | 0.945(0.919, 0.971) | | 0.995(0.979, 1.010) | | 1.006(0.994, 1.018) | | 0.980(0.972, 0.989) | |  | 2.4E-03 | | | 0.699 | |  |
| Ischemic heart disease | 1.065(0.947, 1.199) | | 1.038(0.998, 1.081) | | 1.031(1.007, 1.056) | | 0.993(0.976, 1.010) | |  | -3.0E-02 | | | 0.029 | |  |
| Heart failure | 0.963(0.914, 1.014) | | 0.970(0.943, 0.998) | | 0.988(0.967, 1.010) | | 0.955(0.936, 0.975) | |  | -1.3E-02 | | | 0.275 | |  |
| Heart rhythm disturbances | 1.044(0.782, 1.393) | | 1.184(1.064, 1.317) | | 1.081(1.004, 1.165) | | 0.986(0.933, 1.043) | |  | -4.7E-02 | | | 0.209 | |  |
| Cerebrovascular diseases | 0.909(0.859, 0.962) | | 0.960(0.926, 0.995) | | 1.001(0.975, 1.028) | | 0.992(0.972, 1.013) | |  | 3.3E-02 | | | 0.019 | |  |
| Stroke | 0.905(0.851, 0.962) | | 0.950(0.916, 0.985) | | 1.003(0.976, 1.032) | | 0.990(0.969, 1.011) | |  | 3.5E-02 | | | 0.015 | |  |
| Peripheral vascular disease | 1.026(0.926, 1.136) | | 1.052(1.011, 1.093) | | 1.053(1.021, 1.087) | | 1.011(0.993, 1.028) | |  | -1.3E-02 | | | 0.374 | |  |
| All respiratory diseases | 1.078(1.049, 1.107) | | 1.036(1.018, 1.055) | | 1.022(1.009, 1.034) | | 1.020(1.008, 1.031) | |  | -2.5E-02 | | | <0.001 | |  |
| COPD | 1.009(0.905, 1.125) | | 1.045(1.004, 1.088) | | 1.038(1.010, 1.068) | | 1.047(1.025, 1.069) | |  | 1.7E-02 | | | 0.305 | |  |
| Asthma | 1.042(0.995, 1.091) | | 1.025(0.983, 1.068) | | 0.960(0.927, 0.994) | | 0.952(0.916, 0.988) | |  | -6.3E-02 | | | <0.001 | |  |
| Pneumonia | 1.121(1.081, 1.163) | | 1.055(1.031, 1.079) | | 1.043(1.024, 1.062) | | 1.039(1.023, 1.055) | |  | -3.1E-02 | | | 0.001 | |  |
| Diabetes | 1.051(0.974, 1.133) | | 1.052(1.009, 1.097) | | 1.059(1.024, 1.095) | | 1.033(1.008, 1.059) | |  | 2.6E-03 | | | 0.878 | |  |
| Renal diseases | 1.066(1.006, 1.131) | | 1.109(1.075, 1.144) | | 1.059(1.032, 1.086) | | 1.045(1.027, 1.063) | |  | -2.2E-02 | | | 0.073 | |  |
| Mental health conditions | 1.180(1.013, 1.373) | | 1.141(1.078, 1.207) | | 1.069(1.029, 1.110) | | 1.056(1.028, 1.085) | |  | -4.8E-02 | | | 0.034 | |  |
| Neoplasms | 1.101(1.051, 1.154) | | 1.008(0.986, 1.032) | | 1.020(1.003, 1.036) | | 0.997(0.986, 1.008) | |  | -2.8E-02 | | | 0.001 | |  |
| Heat illness | 1.283(1.068, 1.542) | | 1.284(1.184, 1.393) | | 1.269(1.191, 1.353) | | 1.309(1.244, 1.378) | |  | -9.5E-04 | | | 0.980 | |  |
| Note: RR, relative risk; CI, confidence interval; COPD, Chronic Obstructive Pulmonary Disease. In the meta-regression, we modelled the 1,814 city-specific effect estimates against the city-level log(GDP per capita). The coefficient represented the change in log(RR) associated with an inter-quartile increase in log(GDP per capita). | | | | | | | | | | | | | | |  |

| **Table I**. The number of hospitalization due to specific causes during 2000-2015 hot seasons among elderly people (≥60 years) | | | |
| --- | --- | --- | --- |
|  | **No. of cases, n(%)** | | |
|  | **Lower middle income** | **Upper middle income** | **High income** |
| **All cardiovascular diseases** | 643,707(29.8) | 1,737,404(29.1) | 361,009(29.1) |
| Heart failure | 223,732(10.4) | 484,803(8.1) | 77,487(6.2) |
| Cerebrovascular diseases | 152,176(7.0) | 394,798(6.6) | 79,373(6.4) |
| Heart rhythm disturbances | 12,837(0.6) | 59,071(1.0) | 12,028(1.0) |
| Ischemic heart disease | 77,998(3.6) | 348,605(5.8) | 87,424(7.0) |
| Peripheral vascular disease | 49,767(2.3) | 214,704(3.6) | 46,528(3.7) |
| other cardiovascular diseases | 127,197(5.9) | 235,423(3.9) | 58,169(4.7) |
| **Respiratory diseases** | 388,869(18.0) | 876,797(14.7) | 152,785(12.3) |
| **Neoplasms** | 130,776(6.1) | 571,377(9.6) | 143,557(11.6) |
| **Mental health conditions** | 46,521(2.2) | 475,309(8.0) | 54,686(4.4) |
| **Diabetes** | 89,361(4.1) | 163,477(2.7) | 29,905(2.4) |
| **Renal diseases** | 56,200(2.6) | 159,831(2.7) | 33,623(2.7) |
| **Other diseases** | 804,494(37.2) | 1,981,411(33.2) | 466,805(37.6) |

**Fig G.** The association between heat exposure (every 5℃ increase in daily mean temperature) and all-cause hospitalization, after excluding cities with population size larger than the 95th percentile of 1,814 cities.

Note: CI=confidence interval; **p*-values for difference tested the difference in relative risks between subgroups, estimated by meta-regression. Q1-Q4 represent four quartiles from the lowest to the highest. GDP per capita classifications were based on the World Bank’s 2015 standard. The P-values were derived from the meta-regressions modelling the 1,723 city-specific effect estimates against the city-level literacy rate, urbanization rate, average household income, and log(GDP per capita) separately.


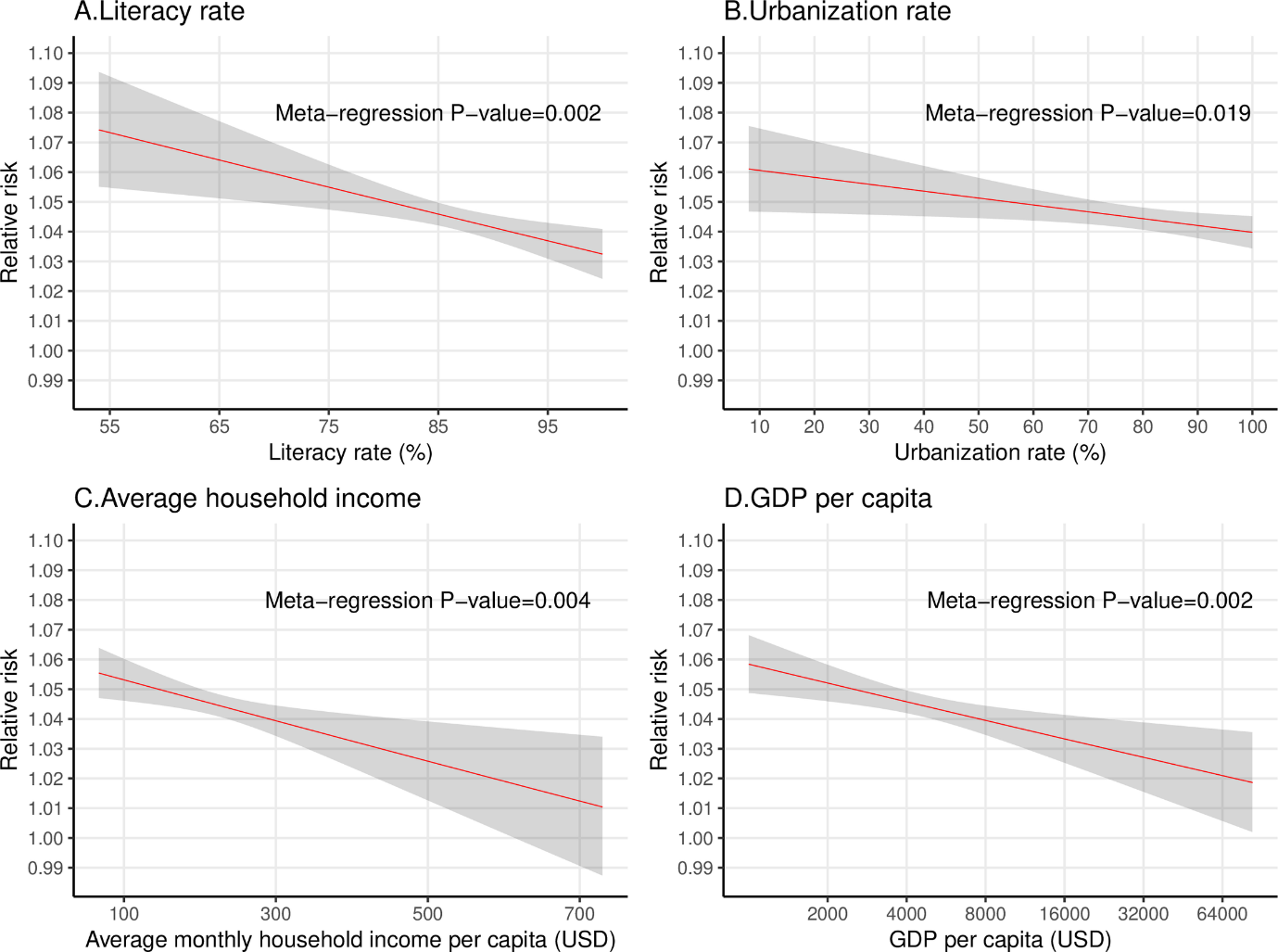


**Fig H.** The relationship between city-level socioeconomic factors and the magnitude of heat-hospitalization association among 1,723 cities, after excluding cities with population size larger than the 95th percentile of 1,814 cities.

Note: relative risk (RR) represents the association between heat exposure (every 5℃ increase in daily mean temperature) and hospitalization during lag 0-7 days. The shadowed area represents 95% confidence intervals. GDP, gross domestic product. USD, United States Dollar. The relationship between RR and four socioeconomic indicators were fitted separately by meta-regression, adjusting for city-specific mean temperature, temperature range, and the ratio of elderly population (≥60 years) and young population (0-19 years). The RRs in the Figs were estimated as the values when city-specific mean temperature, temperature range, and the ratio of elderly population and young population were at average level of 1,723 cities. The x-axis of Fig D was is in log scale, because we added log (GDP per capita) rather than GDP per capita to the meta-regression model.


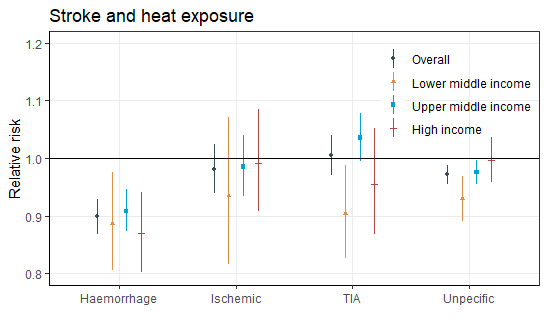


**Fig I.** The association between heat exposure (every 5℃ increase in daily mean temperature) and hospitalization due to different types of stroke during lag 0-7 days, stratified by GDP per capita classifications.

Note: GDP, gross domestic product. GDP per capita classifications were based on the World Bank’s 2015 standard. We classified stroke as haemorrhage stroke (ICD-10 code: I61, I62), ischemic stoke (ICD-10 code: I63), transient ischemic attack (TIA, ICD-10 code: G45). We did not report the result for central retina artery occlusion (ICD-10 code: H34.1^1^) due to its limited number of cases (129 cases).

**References**

1. Kokotailo RA, Hill MD. Coding of stroke and stroke risk factors using International Classification of Diseases, revisions 9 and 10. Stroke 2005;36:1776-81.
