# Supplementary material for: Socioeconomic level and associations between heat exposure and all-cause and cause-specific hospitalization in 1,814 Brazilian cities: A nationwide case-crossover study
Source: PLoS Med. 2020 Oct 8;17(10):e1003369. doi: 10.1371/journal.pmed.1003369 (PMC7544074; doi:10.1371/journal.pmed.1003369)
Supplement: Attachment — Submitted filename: Response to reviewers.docx [file pmed.1003369.s004.docx]

Clare Stone, PhD,

Acting Chief Editor,
PLOS Medicine

Dear Dr. Stone and colleagues,

Thank you very much for your letter on June 25th, 2020. We have carefully revised the paper according to the comments from the editor and reviewers. We have uploaded both the clean version and the marked-up manuscript as required. The responses to the editor’s and reviewers’ comments are detailed as below.

Best regards,

Yuming Guo on behalf of the co-authors

Professor of Global Environmental Health and Biostatistics

Head of Climate, Air Quality Research Unit

Monash University, Melbourne, Australia

**Response to the editor**
**Requests from the editors:**
*Please structure the abstract using the PLOS Medicine headings (Background, Methods and Findings, Conclusions - "Methods and Findings" should be a single subsection heading).
**[Response] Done.**

*In the last sentence of the Abstract Methods and Findings section, please describe the main limitation(s) of the study's methodology.
**[Response] We have added the limitation in the abstract as required (lines 46-48, page 2-3 of marked-up manuscript).**

*At this stage, we ask that you include a short, non-technical Author Summary of your research to make findings accessible to a wide audience that includes both scientists and non-scientists. The Author Summary should immediately follow the Abstract in your revised manuscript. This text is subject to editorial change and should be distinct from the scientific abstract. Please see our author guidelines for more information: <https://journals.plos.org/plosmedicine/s/revising-your-manuscript#loc-author-summary>
**[Response] We have added an Author Summary, please see page 4 of the marked-up manuscript.**

*Please clarify if the analytical approach used in this paper corresponded to one set out in a prospectively-developed protocol or analysis plan? Please state this (either way) early in the Methods section.
a) If a prospective analysis plan (from your funding proposal, IRB or other ethics committee submission, study protocol, or other planning document written before analyzing the data) was used in designing the study, please include the relevant prospectively written document with your revised manuscript as a Supporting Information file to be published alongside your study, and cite it in the Methods section. A legend for this file should be included at the end of your manuscript.
b) If no such document exists, please make sure that the Methods section transparently describes when analyses were planned, and when/why any data-driven changes to analyses took place. 
c) In either case, changes in the analysis-- including those made in response to peer review comments-- should be identified as such in the Methods section of the paper, with rationale.
**[Response] We have attached our prospectively-developed analysis plan, including the modifications in response to reviewers’ comments. Please see the S1 Text.**

*It's notable that the authors acknowledge as a limitation, and the reviewers note, that associations reported in the paper correspond to an ecological analysis, ie at the level of city, not at the level of individual. However, as noted by reviewers, the writeup in the paper doesn't always take this into account and at times slips into claiming effects at the level of the individual - more care should be taken around this. In addition, it would be good to more explicitly state in the abstract the ecological analysis ie that associations are at the level of the city.
**[Response] We have carefully revised our statements throughout the manuscript to avoid inferences about individuals and make it clear that we are evaluating inter-city socioeconomic disparities. Please see our marked-up manuscript at lines 37-52, page 2-3; line 105, page 6; lines 333-337, line 341, page 21; line 415, page 24; lines 404-405, lines 418-419, page 24; lines 456-457, page 25; lines 473-476, page 26. We have also added this limitation to the abstract (lines 46-48, page 2-3 of the marked-up manuscript).**

*Currently the limitations section of the Discussion is quite brief and this doesn't say anything about the potential for effects to be explained by confounding due to unmeasured variables, and this could also be considered/discussed.
**[Response] We have added some detailed discussion of this issue in the limitations section, please see lines 459-471, page 25-26.**

**Response to the reviewers:**
**Reviewer #1:**

I confine my remarks to statistical aspects of this paper. 
I am concerned with the whole city-by-city method. It's not that you can't do analysis on a city basis, but you cannot draw inferences about individuals from city level data - this is known as the ecological fallacy. So, for example, in the first paragraph of the discussion, you cannot say that it was worse for people of a particular age, only for cities with particular age distributions. Throughout the discussion, the authors make statements about individuals that are not justified.

(The ecological fallacy is really counter-intuitive and easy to fall into).
**[Response] Thanks for your comments and suggestions. We have carefully revised our statements throughout the manuscript to avoid inferences about individuals and make it clear that we are evaluating inter-city socioeconomic disparities, rather than intra-city or individual-level disparities. Please see our marked-up manuscript at lines 37-52, page 2-3; line 105, page 6; lines 333-337, line 341, page 21; line 415, page 24; lines 404-405, lines 418-419, page 24; lines 456-457, page 25; lines 473-476, page 26..**

On a more mundane level, the authors should not categorize the independent variables. In *Regression Modeling Strategies* Frank Harrell lists 11 problems with doing this and sums up "nothing could be more disastrous". Leave the various rates as continuous variables and use a spline to investigate nonlinearity.  (It is OK to graph quartiles, but they should not be used for modeling.)
**[Response] We agree with your comments, and have revised the methods of performing meta-regression. Please see the updated Fig 2, Fig S3, S4-S8 Table.**

**We have also tried to use a natural cubic spline to capture the potential non-linearity, but the Bayesian information criterion (BIC) values of non-linear models were generally larger than or close to the BIC values of linear meta-regression model (see S2 Table). Therefore, we kept using linear meta-regression model throughout the analyses. Please see our descriptions about this at lines 193-199, page 9.**

------------------------------------------------------
**Reviewer #2:**

This is a well written paper that should be an important contribution to the literature.  I have a few comments that could improve this paper.
A couple of broadly applicable comments are as follows; with examples; additional line by line comments appear later.
The paper is overall very clearly written. However, some minor grammatical issues appear throughout that suggest a review by a native English speaker could be useful.

**[Response] Thanks for all your great comments. The authors including a native English speaker, have read the manuscript carefully and tried our best to minimize grammatical issues.**

For example, in the abstract, page 2, lines 23 and 24, the word 'the' is not needed before the word 'vulnerability'.

**[Response] We have deleted “the” (lines 23, page 2 of the marked-up manuscript).**

Second, these authors at times use the phrase 'due to' (page 2, line 39) which implies causality. I suggest replacing 'due to' in this instance with 'associated with'; the same substitution is recommended in other places.

**[Response] We agree that we should not assume causality between heat exposure and hospitalization, and we have carefully checked the whole manuscript to avoid any overstatement of causality.**

**However, in the “hospitalisation due to certain diseases” (line 40 of the marked-up manuscript), the “due to” means the attribution of hospitalization to specific diseases, not the causality between heat exposure and hospitalization. Because these diseases were the primary diagnosis of the hospitalizations (see our description in the Data collection in Methods), it would still be appropriate to assume some causality between the diseases and hospitalization. Therefore, it should not be a problem to use “due to” under these circumstances.**

Additional comments are here:
Page 2, lines 39-42; why no confidence intervals?

**[Response] We have added the 95%CIs, please see lines 38-45, page 2, and lines 293-304, page 18.**

Page 2, line 43, disparity should be plural, and stratified should be 'stratifying'

**[Response] Done.**

Page 3, line 46, here again, suggest replacing due to with 'associated with'.

**[Response] Please see our reply above.**

Page 3, line 47, spell out GDP at first use.

**[Response] Done.**

Page 5 lines 93-4. Were secondary diagnoses available and if so why were they not used in classification?  Please explain this.

**[Response] Unfortunately secondary diagnoses were not available. The reason is that the hospitalization data from Brazilian Unified Health System were designed for claiming costs from the government which only require primary diagnoses.**

Page 5 line 95 why is the term 'also' included?

**[Response] We have deleted the “also”.**

Page 5 line 101: were exemptions also granted for Brazilian authors or did they not have access to the data? Presumably someone had to process the data with individual identifiers before it was ready for analysis.

**[Response] Yes, the exemptions also granted for Brazilian authors, because when the data were provided by the Brazilian Ministry of Health, the individual identifiers had already been removed. Those who have access to the individual identifiers did not take part in our study and are not named authors.**

Page 6 line 198; it would be clearer to say '16-year-average' as age is described just previously.
**[Response] Done.**

Page 7 line 7, should say 'remained', not 'retained.'

**[Response] Done.**

Page 8, lines 158- 163.  I can understand why authors may not want to evaluate the city characteristics they describe; (air quality, vegetation, land cover, built environment, health status), but I don't find the reasoning at all convincing. On what basis are they expected to be 'affected by city level SES rather than the converse?'  Why would this be the case in Brazil, by what mechanism? If data on these characteristics are available,I would think it possible to include them in the manner done in a recent related article, Gronlund CJ, Berrocal VJ, White-Newsome JL, Conlon KC, O'Neill MS. Vulnerability to extreme heat by socio-demographic characteristics and area green space among the elderly in Michigan, 1990-2007. Environ Res. 2015;136:449-61.
This section is my reason for suggestion 'major revision' because the section and then later discussion about the interplay between heat exposure and SES just seems too 'facile' and surface level. Even the comment later about the area-level SES variables being correlated thus hampering interpretation would be better illustrated quantitatlively.

**[Response] We did not adjust for those factors mainly because of unavailability of relevant data, particularly for air pollution. The number of air quality stations are very limited in Brazil (see** [**https://waqi.info/#/c/-17.496/-42.618/2.7z**](https://waqi.info/#/c/-17.496/-42.618/2.7z)**). To avoid confusion, we have deleted this sentence in the Methods, and instead discuss this as a limitation in the Discussion (please see lines 459-471, page25-26).**

**As for the correlation between SES indicators, it is hard to find a suitable quantitative method to solve this problem of co-linearity. As we have discussed (see lines 454-457 page 25), although the correlations would make it difficult to identify and compare the independent modifying effect of each SES indicator, it did not affect the interpretation of our main findings. Results based on different SES indicators were similar, and all suggested the existence of inter-city socioeconomic disparities in the heat-hospitalization associations. Therefore, we don’t think it would be necessary to distinguish effects of different SES indicators.**

Page 8 line 173, hospitalisitions should be plural.

**[Response] Done.**

Page 9 Table 1: Why not just spell out Number in lines 1 and 3?

**[Response] Done.**

Why not just say percent instead of proportion in the 3rd and fourth from last lines?

**[Response] We have revised “proportion” to “percentage” (see Table 1).**

Page 10, line 186, correct spelling of characteristics;

**[Response] Done.**

Line 188, take out the word OF (last word)

**[Response] Done.**

Page 11, Figure 2:
I understand why the 'urbanization' is shown from lowest to highest quartile as the 'dose response is similar for this variable as the other ones, but wouldn't the original hypothesis potentially have been that higher urbanization would be linked to greater vulnerability?  Just wonder if the authors might consider presenting those variables a bit differently. (minor point)

**[Response] Because there is no consensus on the cut-off values for defining urban or rural based on urbanization rate, stratifying the urbanization rate into four quartiles was the most straightforward and appropriate way to display results of urbanization. This way of displaying results was also good enough to test the original hypothesis—high urbanization might increase heat vulnerability through urban heat island effect, although our results tend to reject this hypothesis (Please see our detailed discussion in the third and fourth paragraph of Discussion).**

Page 13 line 223, stratified should read 'stratifying'.
**[Response]Done.**

Page 15, paragraph beginning at line 260 needs revision; how do these mechanisms relate to the earlier comment about SES and land cover, built environment, etc?

**[Response] The second paragraph of discussion focused on the potential pathways through which city-level SES affected heat vulnerability. According to the previous literature, there were three important aspects of heat vulnerability: heat exposure, sensitivity, and adaptation capacity [1]. We thus discussed potential important factors contributing to the inter-city socioeconomic disparities for each aspect. This logical framework might be helpful for readers to understand our discussion.**

**Although we agree that factors such as land cover, built environment, and air pollution are important factors that may affect heat vulnerability, they are not likely to be the main contributors to the inter-city socioeconomic disparities. Therefore, we chose not to discuss them in this paragraph. Instead, we have now added some detailed discussion of those unadjusted factors in the limitations section (lines 459-471, page25-26)**

In addition, where are the data on Brazil-specific prevalence of and access to air conditioning?  All the citations are to large multi city studies.  What is known about this and how much AC access and use differ by location, SES, etc.?  This is an omission and needs to be rectified.  There have to be some data that the Brazilian coauthors could help locate, or if not, talk about the limitations and what is known.

**[Response] Thanks for suggestion. We found some data for the household access to air-conditioning in Brazil, but the most recent city-level data were from the 2000 Brazil census. The data generally suggested that the adoption rate of air-conditioning was highest for wealthy, urban households in warm cities[2]. In 2010, Brazil’s national adoption rate of air-conditioner was estimated to be 11.8% (up from 7.4% in 2000), with the lowest adoption rate (6.1%) seen in the Northeast region (the hottest, poorest and least urbanized region in Brazil)[2]. Please see our descriptions added at lines 352-356, page 21.**

Page 15, line 277: the comment on urbanization and how those results differ from results in Vietnam deserves further expansion.

**[Response] We have expanded this paragraph to discuss and explain the difference between our study and the Vietnamese study in detail. Please see lines 366-391, pages 22-23.**

Page 16, lines 284-7.  These sentences with a recommendation do not seem well supported; why would a heat exposure vulnerability index in particular relate to the contradictory findings of this article from the Lancet recommendation?  Needs revision.

**[Response] The Lancet report only treated urbanization as a surrogate of urban heat island effect, but did not consider the socioeconomic improvements along with urbanization. Therefore, the Lancet report made an inappropriate recommendation to simply treat high urbanization as an index of high heat vulnerability, which was not true in our study or two previous studies [3, 4]. We have added more discussion to make this point clearer, please see lines 377-391, pages 22-23.**

Page 17, line 313; should read 'driver'

**[Response] Done.**

Page 17, line 321, remove the phrase 'there are also'.

**[Response] Done.**

Page 18, lines 339-340: were the individual level data not available on the hospitalization records?  It is worth describing whether this is the case or not

**[Response] The hospitalization dataset did not have any information for the patients’ individual level socioeconomic indicators. To make this clearer, we have revised this sentence to (line 432, page25):**

**“Due to the unavailability of individual level socioeconomic data in the hospitalization records, we had to rely on city-level socioeconomic data, which could not evaluate the intra-city or individual-level socioeconomic inequality.”**

-----------------------------------------------------------
**Reviewer #3:**

This is an interesting study on a very important topic, namely that of inequalities in climate impacts. However, I do have serious reservations with the design of the study:

-       My first issue is rather fundamental, and is related to the scale of the study. The authors have aggregated climate as well as socio-economic indicators on a city level. For temperature this can be defended, even though there is likely to be variation in temperature within cities. However, for socio-economic levels, I don't see this as a valid approach. The variation, especially in large cities such as Sao Paolo or Rio de Janeiro, is enormous, and using one value, e.g. average household income, to define all inhabitants seems very flawed.

**[Response] We have acknowledged that our analyses based on city-level average socioeconomic indicators cannot evaluate the individual-level and intra-city socioeconomic disparities (line 447-448, page 25 of the marked-up manuscript). We have claimed throughout the manuscript that we aimed to evaluate inter-city socioeconomic disparities. As city-level socioeconomic indicators (e.g., urbanization rate, average household income) reflect the city’s characteristics, it is appropriate to use them to evaluate inter-city socioeconomic disparities. This approach has been validated in several previous studies in terms of capturing inter-city (or inter-region) variation in mortality or morbidity impacts of heat exposure[4-10].**

**We agree that the socioeconomic variation within a big city could be enormous. However, most cities included in our analyses had small population sizes (see Fig S1 below and in supplement), thus most of them were small cities. For example, 87% of the 1,814 cities had a population size less than 100,000. The intra-city socioeconomic variations within those cities tended to be small given the small population sizes. We performed a sensitivity analysis by excluding cities with population size larger than the 95th percentile of 1,814 cities, and the results changed minimally compared to our main analyses (please see Fig S7-S8). Please see our added descriptions about these issues in the Methods (lines 124-127, page 6; lines 215-219, page 10) and Results (lines 316-318, page 20) section.**

**Fig S1. The distribution of the 16-year average population size for 1,814 cities**

**Notes: P95 represents the 95th percentile, which corresponded to 243,271 in this figure.** **The x-axis is on log scale (1e+04 = 10,000; 1e+05 = 100,000 etc).**

-       Furthermore, for some indicators, only one or two data points were available, and those were interpolated to 15 years. This is a very inaccurate way of dealing with indicators that are already not very representative for a large share of city inhabitants.

**[Response] We acknowledge the limitation of the data gap for certain indicators, including urbanization rate (2000 and 2010 census data), literacy rate (2000 and 2010 census data), household income (only 2010 census data). We only performed interpolation for urbanization rate and literacy rate, not for household income. According to data from the World Bank, the national urbanization rate of Brazil increased from 81.2% in 2000 to 85.8% in 2015 (**<https://data.worldbank.org/indicator/SP.URB.TOTL.IN.ZS?locations=BR>**), and the national literacy rate increased from 86.4% to 92.0% (**

<https://data.worldbank.org/indicator/SE.ADT.LITR.ZS?locations=BR>). **Given the generally small long-term changes, the errors of linear interpolation should be minimal.**

**As the city-specific effect estimates were based on 16-year data, using the 16-year average socioeconomic indicators is the most appropriate approach to capture the inter-city socioeconomic disparities. Therefore, linear interpolation for urbanization and literacy rates is suitable to utilize all available data.**

**Last but not the least, we have city-level GDP per capita for every year during 2000-2015, and GDP per capita was highly correlated with other socioeconomic indicators. The results stratifying by GDP per capita were generally consistent with results stratifying by urbanization rate, household income and literacy rate (Fig2-4, Fig S4-S6). These four socioeconomic indicators could verify each other in detecting the inter-city socioeconomic disparities in heat-hospitalization association. Therefore, the data gaps and interpolation errors were not likely to affect our main findings.**

-   In addition, temperature is not independent of socio-economic indicators, as the authors also indicate that cities in the South are wealthier and colder. This also leads to spurious interpretation of the findings, which occurs in various places in the Results and Discussion sections

**[Response] In Fig 5, we have performed meta-regression adjusting for city-level mean temperature and temperature range. The results suggest that the inter-city socioeconomic disparities in heat-hospitalization association remained significant after excluding confounding effect of temperature.**

In summary, I think the study would be publishable if the focus is only on the relationship between temperature and hospitalization, which by itself is already rather innovative. But I would remove the socio-economic component completely (second stage of the analysis), which is very inaccurate.
**[Response] Our study adds value to the current literature.**

**First, we have provided the overall effect estimates (i.e., not stratified by socioeconomic status) of heat-hospitalization association for both all-cause and cause-specific hospitalization (Fig 2-4; S4 Table). This could give readers a comprehensive view of the heat-related morbidity, and the results tended to be robust given our huge nationwide dataset.**

**Second, our analyses about socioeconomic disparities have important public health implications despite some limitations. We have acknowledged that our analyses cannot evaluate individual-level and intra-city inequality. However, the findings about the inter-city socioeconomic disparities could still be quite relevant for adaptation policy at city, state or national level for promoting health equity under global warming. Promoting health equity between cities is an indispensable part of promoting within-country health equity, one of priorities of the Sustainable Development Goals (SGDs)(line 453, page 25) [11].**

**As we proved above, the 16-year average socioeconomic indicators are accurate measures of city average socioeconomic situation, given the small population sizes of most cities and the slow long-term socioeconomic changes in Brazil. The results based on four different socioeconomic indicators (GDP per capita, urbanization rate, household income and literacy rate) have verified each other. This further supports the robustness of our findings about the inter-city socioeconomic disparities in heat-hospitalization association.**

I also have some smaller comments:
Methods:
-       The authors refer on several occasions to methodological aspects that have been described in previous publications. However, this is not sufficient for someone reading this publication by itself, and there should not be a necessity to look for additional sources.

**[Response] We have added more details about the first-stage statistical analysis, including the model equations (lines 167-176, page 8). After adding these details, we believe that readers don’t need to read additional sources in order to understand this paper.**

Results:

-       Line 169: what does this number of hospitalisations represent? Is this only for the warmest four months, as mentioned in the methods?

**[Response] Yes, only for hot season. We have revised it to:**

**“A total of 49,145,997 hospital admissions (58.5% female) from 1,814 Brazilian cities in the hot season between 2000 and 2015 were included in the analyses”**

-       Lines 175-177: this sentence is confusing, consider revision and clarification on what the mean and median refer to (within cities, between cities; within years, between years).

**[Response] We have added a footnote beneath the Table 1 to clarify this:**

**“For daily mean temperature, the median (IQR), min (minimum) and max (maximum) were based on all city-specific daily observations for the 1,814 cities in the hot season between 2000 and 2015.”**

-       Line 192-193: 5 °C increase above what? There should be a baseline temperature.

**[Response] Because the relationship was linear (see Statistical analyses, line 181, page 8), so there was no baseline temperature for the association. However, we have to acknowledge that the linear relationship only applies to the hot season, so we have revised this sentence to:**

**“we estimated that every 5℃ increase in daily mean temperature in the hot season was associated with 4.0%”**

-       There is no description of the results on the relationship between temperature and cause-specific hospitalization (independent of socio-economic indicators). This would be a very interesting addition.
**[Response]** **We have added the effect estimates for all cities combined (i.e., without stratification by socioeconomic status), please see the description at line 281-291, page 18 and the updated Fig 3, Fig 4 and S4 Table.**

**Reference**

1.	Romero-Lankao P, Qin H, Dickinson K. Urban vulnerability to temperature-related hazards: A meta-analysis and meta-knowledge approach. Global Environmental Change. 2012;22(3):670-83. doi: 10.1016/j.gloenvcha.2012.04.002.

2.	Forrester S. Residential Cooling Load Impacts on Brazil's Electricity Demand [M.Sc. Thesis]. Ann Arbor, Michigan, USA: University of Michigan; 2019.

3.	Hu K, Guo Y, Hochrainer-Stigler S, Liu W, See L, Yang X, et al. Evidence for Urban–Rural Disparity in Temperature–Mortality Relationships in Zhejiang Province, China. Environmental health perspectives. 2019;127(3):037001. doi: 10.1289/EHP3556.

4.	Chen K, Zhou L, Chen X, Ma Z, Liu Y, Huang L, et al. Urbanization Level and Vulnerability to Heat-Related Mortality in Jiangsu Province, China. Environmental health perspectives. 2016;124(12):1863-9. Epub 2016/05/07. doi: 10.1289/EHP204. PMID: 27152420

5.	Phung D, Guo YM, Nguyen HTL, Rutherford S, Baum S, Chu C. High temperature and risk of hospitalizations, and effect modifying potential of socio-economic conditions: A multi-province study in the tropical Mekong Delta Region. Environ Int. 2016;92-93:77-86. doi: 10.1016/j.envint.2016.03.034.

6.	Vicedo-Cabrera AM, Gasparrini A, Armstrong B, Sera F, Guo Y, Hashizume M, et al. How urban characteristics affect vulnerability to heat and cold: a multi-country analysis. International journal of epidemiology. 2019. doi: 10.1093/ije/dyz008.

7.	Curriero FC, Heiner KS, Samet JM, Zeger SL, Strug L, Patz JA. Temperature and mortality in 11 cities of the eastern United States. Am J Epidemiol. 2002;155(1):80-7. PMID: 11772788.

8.	O'Neill MS, Zanobetti A, Schwartz J. Modifiers of the temperature and mortality association in seven US cities. Am J Epidemiol. 2003;157(12):1074-82. doi: 10.1093/aje/kwg096.

9.	Hattis D, Ogneva-Himmelberger Y, Ratick S. The spatial variability of heat-related mortality in Massachusetts. Applied Geography. 2012;33:45-52. doi: 10.1016/j.apgeog.2011.07.008.

10.	Medina-Ramon M, Schwartz J. Temperature, temperature extremes, and mortality: a study of acclimatisation and effect modification in 50 US cities. Occup Environ Med. 2007;64(12):827-33. doi: 10.1136/oem.2007.033175.

11.	Hosseinpoor AR, Bergen N, Magar V. Monitoring inequality: an emerging priority for health post-2015. Bull World Health Organ. 2015;93(9):591-A. doi: 10.2471/BLT.15.162081. PMID: 26478619.
